# Supplementary material for: True prevalence of long-COVID in a nationwide, population cohort study
Source: Nat Commun. 2023 Nov 30;14:7892. doi: 10.1038/s41467-023-43661-w (PMC10689486; doi:10.1038/s41467-023-43661-w)
Supplement: Supplementary file 1 — Supplementary information [file 41467_2023_43661_MOESM1_ESM.pdf]

Supplementary Figure 1. Self-completed Questionnaire

Q1. Have you ever had a positive Covid-19 test? (yes/no)

Q2. Have you had a Covid-19 vaccine? (yes/no)

Q3. What Covid-19 symptoms did you have? [only if Q1 yes] (select all that apply)
Fever; Cough; Change in taste; Change in smell; Tiredness (fatigue); Headache; Ear pain; Runny nose; Muscle aches or weakness; Joint pain; Breathlessness; Chest pain; Sore throat; Hoarse voice; Loss of appetite; Stomach (abdominal) pain; Diarrhoea; Confusion; Seizures; Hair loss; Unconscious/semi-conscious; I didn't have any symptoms [go to Q5]

Q4. How long did your symptoms last?
Less than 1 week;1-4 weeks; More than 4 weeks

Q5. How do you feel now?
Fully recovered; Partly recovered; Not recovered

Q6. Before your Covid-19 test, did you have any health issues or medical conditions?
Yes; No [go to Q8]

Q7. Which health issues or medical conditions did you have? (select all that apply)
Arthritis; Asthma/bronchitis/COPD; Cancer; Cystic fibrosis; Deep vein thrombosis; Depression/anxiety; Diabetes; Heart disease; Heart failure; High blood pressure; HIV; Home oxygen; Kidney disease; Liver disease; Neurological condition; Overweight; Obese; Pulmonary embolism; Pulmonary fibrosis; Stroke; I didn't have any of these [go to Q8]

Q8. Have you experienced any of the following in the last week? (select all that apply)
Dry cough; Cough with phlegm; Change in taste; Change in smell; Problems hearing; Problems with eyesight; Tired; Headache; Muscle aches/weakness; Pins and needles; Joint pain; Breathless; Chest pain; Palpitations; Poor appetite; Stomach (abdominal) pain; Feeling sick/vomiting; Diarrhoea; Constipation; Weight loss; Anxious/depressed; Confusion/difficulty concentrating; Dizzy/blackouts/fits; Balance problems; Skin rash; Problems sleeping; I haven't experienced any of these [go to Q10]

Q9. Are any of these new or worse since your Covid-19 test? (select all that apply)
Response list from Q8; None of these are new or worse [go to Q10]

Q10. Is your current health causing you difficulty with any of the following? Select all that apply.
Walking/getting around; Housework/DIY/chores; Working/studying; Washing/dressing; Exercise/sports; Hobbies; Relationships; None of these apply [go to Q12; if also no to Q2 go to Q13]

Q11. Which of the difficulties you're experiencing are new/worse since having your Covid-19 test? (select all that apply)
Response list from Q10; None of these are new or worse [go to Q13]

Q12. Has your job changed since your Covid-19 test?
No change; Different due to my Covid-19 illness; Different for other reasons; None of these apply

Q13. What is your ethnicity?
White Scottish; White Other British; White Irish; White Gypsy/Traveller; White Polish; Other white ethnic group; Mixed or multiple ethnic groups; Pakistani, Pakistani Scottish or Pakistani British; Indian, Indian Scottish or Indian British; Bangladeshi, Bangladeshi Scottish or Bangladeshi British; Chinese, Chinese Scottish or Chinese British; Other Asian, Asian Scottish or Asian British; African, African Scottish or African British; Other African; Caribbean, Caribbean Scottish or Caribbean British; Black, Black Scottish or Black British; Other Caribbean or Black; Arab, Arab Scottish or Arab British; Other ethnic group; Prefer not to say

Q14. How would you rate your health today?
EQ-5D thermometer (0 Worst health I can imagine – 100 Best health I can imagine)

Supplementary Table 1. Crude and adjusted attributable prevalence of long-COVID at 6, 12 and 18 months following symptomatic SARS-CoV-2 infection

	6 months				12 months				18 months			
	Never infected	Infected			Never infected	Infected			Never infected	Infected		
	Prevalence	Prevalence	Crude attributable prevalence (95% CI)	Adjusted* attributable prevalence (95% CI)	Prevalence	Prevalence	Crude attributable prevalence (95% CI)	Adjusted* attributable prevalence (95% CI)	Prevalence	Prevalence	Crude attributable prevalence (95% CI)	Adjusted* attributable prevalence (95% CI)
**Overall**	50.8	64.5	13.8 (13.2,14.3)	6.6 (6.3, 6.9)	55.0	67.8	12.8 (11.9,13.6)	6.5 (6.0, 6.9)	56.2	72.5	16.3 (14.4,18.2)	10.4 (9.1, 11.6)
**Age group (years)**												
16-29	52.5	64.9	12.4 (11.2,13.5)	5.1 (4.5, 5.7)	56.2	65.9	9.7 (7.6,11.8)	4.1 (3.0, 5.2)	56.9	69.0	12.1 (6.8,17.5)	5.6 (2.0, 9.2)
30-39	52.4	65.8	13.4 (12.1,14.7)	6.5 (5.8, 7.2)	56.1	68.2	12.1 (9.9,14.3)	6.4 (5.3, 7.6)	56.4	71.5	15.2 (9.8,20.5)	11.0 (6.9, 15.1)
40-49	51.5	64.9	13.5 (12.2,14.7)	7.5 (6.9, 8.2)	57.2	70.2	13.0 (11.1,15.0)	8.0 (6.8, 9.1)	58.2	74.2	16.0 (11.5,20.5)	12.0 (8.7, 15.4)
50-59	49.0	65.8	16.8 (15.7,17.9)	8.1 (7.5, 8.7)	54.8	69.2	14.4 (12.8,16.1)	7.5 (6.7, 8.4)	55.7	73.7	17.9 (14.5,21.4)	13.1 (10.8, 15.4)
60-69	48.4	61.5	13.1 (11.7,14.5)	6.6 (5.9, 7.2)	52.0	65.9	14.0 (12.0,16.0)	6.4 (5.4, 7.3)	55.6	73.0	17.4 (13.3,21.5)	8.9 (6.4, 11.4)
70+	51.0	60.2	9.2 (7.1,11.4)	3.3 (2.2, 4.3)	54.2	63.9	9.7 (6.3,13.0)	3.6 (2.1, 5.2)	54.8	70.0	15.1 (7.3,23.0)	6.0 (2.1, 10.0)
**Sex**												
Female	54.4	68.5	14.1 (13.4,14.8)	7.4 (7.1, 7.8)	59.0	71.5	12.5 (11.4,13.6)	7.0 (6.4, 7.6)	59.9	75.5	15.5 (13.1,18.0)	11.2 (9.4, 12.9)
Male	46.1	58.2	12.1 (11.3,13.0)	5.5 (5.1, 5.9)	49.9	62.0	12.1 (10.8,13.5)	5.7 (5.1, 6.4)	51.5	67.9	16.4 (13.5,19.4)	9.5 (7.6, 11.4)
**SIMD**												
1 (most deprived)	55.7	69.8	14.0 (12.9,15.2)	6.2 (5.6, 6.8)	60.4	72.3	11.9 (10.1,13.6)	5.6 (4.7, 6.5)	61.4	77.8	16.4 (12.6,20.2)	10.0 (7.4, 12.5)
2	52.9	67.2	14.3 (13.2,15.5)	6.7 (6.1, 7.3)	57.2	71.0	13.8 (11.9,15.6)	7.3 (6.4, 8.3)	58.2	75.5	17.3 (13.1,21.4)	11.6 (8.7, 14.5)
3	50.6	64.9	14.3 (13.1,15.5)	7.1 (6.5, 7.8)	55.2	68.2	13.0 (11.1,15.0)	6.6 (5.6, 7.7)	55.9	72.0	16.0 (11.7,20.4)	10.8 (8.0, 13.7)
4	48.5	62.1	13.6 (12.4,14.7)	6.8 (6.2, 7.5)	51.5	65.7	14.2 (12.3,16.1)	7.0 (6.0, 8.0)	54.6	69.7	15.1 (10.7,19.5)	9.6 (6.6, 12.7)
5 (least deprived)	45.9	59.1	13.2 (12.0,14.4)	6.2 (5.5, 6.8)	50.2	61.8	11.6 (9.7,13.5)	5.8 (4.8, 6.9)	49.7	65.7	16.0 (11.5,20.5)	10.0 (6.9, 13.0)
**Number of pre-existing health conditions**												
0	42.2	58.9	16.6 (16.0,17.3)	7.7 (7.4, 8.1)	45.0	62.6	17.6 (16.6,18.7)	8.2 (7.6, 8.7)	43.6	66.7	23.1 (20.6,25.5)	14.0 (12.2, 15.8)
1	61.7	73.3	11.6 (10.3,12.9)	5.3 (4.6, 6.1)	65.5	73.9	8.4 (6.3,10.5)	4.2 (3.0, 5.3)	66.8	78.0	11.2 (6.6,15.8)	5.0 (2.1, 7.8)
2-3	74.9	81.7	6.8 (5.5,8.0)	3.3 (2.6, 3.9)	78.4	83.3	5.0 (3.2,6.7)	2.6 (1.7, 3.5)	78.1	88.3	10.2 (6.5,13.8)	5.3 (3.0, 7.7)
≥4	84.5	88.6	4.1 (2.1,6.2)	1.7 (0.8, 2.7)	86.5	89.4	2.9 (0.0,5.8)	1.7 (0.3, 3.1)	87.9	93.9	6.0 (0.9,11.1)	1.4 (-1.2, 3.9)
**Ethnic group**												
White	52.7	65.5	12.8 (12.2,13.3)	6.8 (6.5, 7.1)	57.1	69.1	12.0 (11.1,12.8)	6.6 (6.2, 7.1)	58.2	73.9	15.7 (13.7,17.6)	10.4 (9.0, 11.7)
South Asian	43.0	58.2	15.2 (10.9,19.5)	6.4 (4.3, 8.6)	45.3	60.7	15.3 (8.2,22.5)	6.3 (2.8, 9.8)	45.3	63.0	17.7 (1.9,33.5)	14.1 (1.3, 26.8)
Black	39.7	58.7	19.0 (12.0,26.0)	8.5 (4.9, 12.0)	42.9	53.6	10.7 (-0.7,22.0)	3.0 (-1.8, 7.8)	69.6	59.5	-10.1 (-34.7,14.5)	-12.9 (-30.6, 4.8)
Other	48.2	63.2	15.0 (11.3,18.7)	6.4 (4.6, 8.1)	49.5	62.9	13.4 (6.7,20.0)	6.5 (3.5, 9.5)	53.9	72.9	19.0 (5.3,32.6)	10.8 (3.3, 18.3)
Missing	28.7	42.5	13.8 (11.3,16.3)	4.0 (3.1, 4.8)	31.2	42.8	11.7 (7.7,15.6)	3.8 (2.3, 5.2)	29.2	47.4	18.2 (9.7,26.6)	9.7 (5.2, 14.1)
**Number of vaccination doses**												
0	51.0	64.2	13.2 (12.4,14.0)	5.1 (4.8, 5.4)	56.0	67.9	11.9 (10.8,13.0)	4.7 (4.3, 5.1)	56.2	72.5	16.3 (14.3,18.2)	10.3 (9.0, 11.6)
1	52.3	66.0	13.8 (11.7,15.9)	6.5 (5.5, 7.6)	55.2	68.9	13.7 (10.4,17.1)	9.5 (7.2, 11.8)	56.4	72.3	15.9 (5.7,26.0)	11.0 (3.4, 18.5)
2	49.5	66.8	17.4 (16.4,18.4)	7.4 (6.9, 8.0)	50.1	67.6	17.4 (15.6,19.2)	10.5 (9.2, 11.8)	51.9	84.0	32.1 (8.4,55.8)	2.8 (-8.7, 14.2)
≥3	51.9	62.6	10.7 (9.4,12.1)	7.2 (6.2, 8.2)	52.3	68.1	15.7 (7.9,23.6)	6.7 (2.5, 10.8)	-	-	-	-
**Variant period**												
Pre VOC	50.8	65.2	14.4 (13.1,15.8)	4.8 (4.4, 5.3)	56.8	69.8	13.0 (11.3,14.8)	4.3 (3.7, 4.9)	56.1	73.3	17.2 (14.5,19.9)	7.6 (6.4, 8.7)

No dominant (1)	51.6	65.5	13.9 (12.6,15.2)	4.1 (3.7, 4.5)	55.8	68.6	12.7 (11.1,14.3)	4.1 (3.6, 4.6)	57.0	72.7	15.7 (12.1,19.3)	12.8 (9.9, 15.6)
Alpha	53.1	66.8	13.7 (11.2,16.2)	3.9 (3.2, 4.6)	56.0	69.8	13.8 (10.1,17.6)	4.0 (3.0, 5.1)	56.2	71.6	15.5 (9.0,21.9)	9.5 (4.7, 14.4)
No dominant (2)	52.4	64.1	11.7 (8.5,15.0)	3.7 (2.6, 4.9)	50.3	69.5	19.3 (13.9,24.6)	9.9 (7.0, 12.8)	51.7	67.9	16.2 (4.3,28.1)	13.7 (4.8, 22.7)
Delta	49.1	66.7	17.6 (16.6,18.6)	6.7 (6.2, 7.1)	50.1	66.8	16.6 (15.0,18.3)	10.4 (9.2, 11.6)	-	-	-	-
No dominant (3)	48.8	62.2	13.4 (11.5,15.3)	8.9 (7.5, 10.2)	52.6	66.7	14.1 (0.6,27.6)	7.0 (-2.5, 16.5)	-	-	-	-
Omicron	52.6	63.3	10.7 (9.2,12.1)	7.9 (6.9, 9.0)	-	-	-	-	-	-	-	-

CI confidence interval; SIMD Scottish Index of Multiple Deprivation; VOC Variant of concern

*Adjusted for age, sex, ethnic group, SIMD quintile, number of pre-existing health conditions, asthma/bronchitis/COPD, CHD, depression/anxiety, diabetes, variant period, Covid-19 severity (hospitalised yes/no), and number of vaccination doses.
