# Supplementary material for: True prevalence of long-COVID in a nationwide, population cohort study
Source: Nat Commun. 2023 Nov 30;14:7892. doi: 10.1038/s41467-023-43661-w (PMC10689486; doi:10.1038/s41467-023-43661-w)
Supplement: Supplementary file 3 — Reporting Summary [file 41467_2023_43661_MOESM3_ESM.pdf]

nature portfolio

Corresponding author(s): Jill P Pell

Last updated by author(s): Nov 8, 2023

Reporting Summary

Nature Portfolio wishes to improve the reproducibility of the work that we publish. This form provides structure for consistency and transparency in reporting. For further information on Nature Portfolio policies, see our Editorial Policies and the Editorial Policy Checklist.

Statistics

For all statistical analyses, confirm that the following items are present in the figure legend, table legend, main text, or Methods section.

n/a	Confirmed	
☐	☒	The exact sample size (*n*) for each experimental group/condition, given as a discrete number and unit of measurement
☐	☒	A statement on whether measurements were taken from distinct samples or whether the same sample was measured repeatedly
☐	☒	The statistical test(s) used AND whether they are one- or two-sided<br>*Only common tests should be described solely by name; describe more complex techniques in the Methods section.*
☐	☒	A description of all covariates tested
☐	☒	A description of any assumptions or corrections, such as tests of normality and adjustment for multiple comparisons
☐	☒	A full description of the statistical parameters including central tendency (e.g. means) or other basic estimates (e.g. regression coefficient) AND variation (e.g. standard deviation) or associated estimates of uncertainty (e.g. confidence intervals)
☐	☒	For null hypothesis testing, the test statistic (e.g. *F*, *t*, *r*) with confidence intervals, effect sizes, degrees of freedom and *P* value noted<br>*Give P values as exact values whenever suitable.*
☒	☐	For Bayesian analysis, information on the choice of priors and Markov chain Monte Carlo settings
☒	☐	For hierarchical and complex designs, identification of the appropriate level for tests and full reporting of outcomes
☒	☐	Estimates of effect sizes (e.g. Cohen's *d*, Pearson's *r*), indicating how they were calculated

Our web collection on statistics for biologists contains articles on many of the points above.

Software and code

Policy information about availability of computer code

Data collection	No software used.
Data analysis	Stata version 16.0.

For manuscripts utilizing custom algorithms or software that are central to the research but not yet described in published literature, software must be made available to editors and reviewers. We strongly encourage code deposition in a community repository (e.g. GitHub). See the Nature Portfolio guidelines for submitting code & software for further information.

Data

Policy information about availability of data

All manuscripts must include a data availability statement. This statement should provide the following information, where applicable:

- Accession codes, unique identifiers, or web links for publicly available datasets
- A description of any restrictions on data availability
- For clinical datasets or third party data, please ensure that the statement adheres to our policy

The datasets (National Health Service Scotland's PCR testing platform, Scottish Morbidity Records 01 and 04, Prescribing Information System, Covid-19 vaccination database, and General Registrar Office death certificates) analysed during the current study are available in the National Services Scotland National Safe Haven, https://www.isdscotland.org/Products-and-Services/eDRIS/Use-of-the-National-Safe-Haven/. This protects the confidentiality of the data and ensures that

Information Governance is robust. Applications to access health data in Scotland are submitted to the NHS Scotland Public Benefit and Privacy Panel for Health and Social Care. Information can be found at https://www.informationgovernance.scot.nhs.uk/pbpphsc/

Research involving human participants, their data, or biological material

Policy information about studies with human participants or human data. See also policy information about sex, gender (identity/presentation), and sexual orientation and race, ethnicity and racism.

Reporting on sex and gender	Total sample size was 198,096, of which 116,513 (59%) were female and 81,583 (41%) male.
Reporting on race, ethnicity, or other socially relevant groupings	91% of participants were white, 1.5% south asian, 0.6% black, 1.9% other, and for 5.0% ethnicity was missing.
Population characteristics	See below.
Recruitment	Adults (>16 years) in Scotland with a positive PCR test for SARS-CoV-2 from April 2020 to May 2022 were invited to participate along with a comparison group who had had a negative test but never had a positive test, matched 3:1 by age, sex, and area-based socioeconomic deprivation quintile. Invitations were sent by automated SMS text message. Additional data were obtained through linkage to electronic health records. Selection bias may be present in those who were tested for SARS-CoV-2, those who completed the questionnaire, and those who consented to linkage. During the time period when index PCR tests were conducted testing was available to everyone free of charge. However, people might be less likely to have been tested if their symptoms were mild resulting in some bias in testing. Furthermore, selection bias in questionnaire completion could potentially lead to overestimation of associations if having ongoing symptoms made participation more likely, or alternatively underestimation of associations if having more severe ongoing symptoms affected the ability to participate. In terms of linkage consent it is difficult to determine what direction of effect this might have.
Ethics oversight	Study approval was obtained from the West of Scotland Research Ethics Committee (ref.170 21/WS/0020) and the Public Benefit and Privacy Panel (ref. 2021-0180).

Note that full information on the approval of the study protocol must also be provided in the manuscript.

Field-specific reporting

Please select the one below that is the best fit for your research. If you are not sure, read the appropriate sections before making your selection.

☐ Life sciences ☒ Behavioural & social sciences ☐ Ecological, evolutionary & environmental sciences

For a reference copy of the document with all sections, see nature.com/documents/nr-reporting-summary-flat.pdf

Behavioural & social sciences study design

All studies must disclose on these points even when the disclosure is negative.

Study description	Nationwide ambidirectional cohort study (quantitative data). An online questionnaire collected information on pre-existing health conditions and 26 current symptoms. Additional data were obtained through linkage to electronic health records - both five years prior their index test and subsequent to the test (up to January 2022) - on hospitalizations (Scottish Morbidity Record 01/04), dispensed prescriptions (Prescribing Information System), vaccinations, and death certificates (General Registrar Office).
Research sample	Adults (>16 years) in Scotland with a positive PCR test for SARS-CoV-2 from April 2020 to May 2022 were invited to participate along with a comparison group who had had a negative test but never had a positive test, matched 3:1 by age, sex, and area-based socioeconomic deprivation quintile. The rationale behind the recruitment method was to collect an unselected general population study sample. Overall response rate was 9%. Participants with previous symptomatic infection were compared with those never infected. Compared with those who did not provide consent, participants in the final sample were more likely to be female (58.8% vs 51.8%; p-value <0.001), were older (>40 years 64.0% vs 51.1%; p-value <0.001) and slightly more deprived (most deprived SIMD quintile 20.8% vs 20.4%; p-value <0.001).
Sampling strategy	There was no predetermined sample size. This is a nationwide study that invited adults in Scotland who had a positive PCR test for SARS-CoV-2 from April 2020 to May 2022 along with a comparison group. The sample comprises the people who responded.
Data collection	A self-completed online questionnaire collected information on pre-existing health conditions at the time of the index test (first positive test or, for comparison group, most recent negative test) as well as current symptoms, limitations in daily activities and quality of life. Those who had tested positive also provided information on symptoms during the initial infection and current recovery status. Questionnaires were completed 6, 12, and 18 months after the index test. Additional data were obtained through linkage to electronic health records both five years prior their index test and subsequent to the test (up to November 2022) on hospitalizations (Scottish Morbidity Record 01/04), dispensed prescriptions (Prescribing Information System), vaccinations, and death certificates (General Registrar Office). Researchers were not blinded to exposure group but nor were they involved in data collection.
Timing	Questionnaire responses were collected between May 2021 and November 2022.

Data exclusions	53,530 participants were excluded because they reported a previous positive test that was not recorded on the database, and 5,715 because they had asymptomatic infections.
Non-participation	Response rate 9%.
Randomization	This is an observational study with group membership determined by Covid test result. Potential confounders were included in multivariate analysis.

Reporting for specific materials, systems and methods

We require information from authors about some types of materials, experimental systems and methods used in many studies. Here, indicate whether each material, system or method listed is relevant to your study. If you are not sure if a list item applies to your research, read the appropriate section before selecting a response.

Materials & experimental systems

n/a	Involved in the study
☒	☐ Antibodies
☒	☐ Eukaryotic cell lines
☒	☐ Palaeontology and archaeology
☒	☐ Animals and other organisms
☒	☐ Clinical data
☒	☐ Dual use research of concern
☒	☐ Plants

Methods

n/a	Involved in the study
☒	☐ ChIP-seq
☒	☐ Flow cytometry
☒	☐ MRI-based neuroimaging

Plants

Seed stocks	*Report on the source of all seed stocks or other plant material used. If applicable, state the seed stock centre and catalogue number. If plant specimens were collected from the field, describe the collection location, date and sampling procedures.*
Novel plant genotypes	*Describe the methods by which all novel plant genotypes were produced. This includes those generated by transgenic approaches, gene editing, chemical/radiation-based mutagenesis and hybridization. For transgenic lines, describe the transformation method, the number of independent lines analyzed and the generation upon which experiments were performed. For gene-edited lines, describe the editor used, the endogenous sequence targeted for editing, the targeting guide RNA sequence (if applicable) and how the editor was applied.*
Authentication	*Describe any authentication procedures for each seed stock used or novel genotype generated. Describe any experiments used to assess the effect of a mutation and, where applicable, how potential secondary effects (e.g. second site T-DNA insertions, mosiacism, off-target gene editing) were examined.*
